# Comparative Prognostic Value of Coronary Calcium Score and Perivascular Fat Attenuation Index in Coronary Artery Disease

**DOI:** 10.3390/jcm13175205

**Published:** 2024-09-02

**Authors:** Maria Teresa Savo, Morena De Amicis, Dan Alexandru Cozac, Gabriele Cordoni, Simone Corradin, Elena Cozza, Filippo Amato, Eleonora Lassandro, Stefano Da Pozzo, Donatella Tansella, Diana Di Paolantonio, Maria Maddalena Baroni, Antonio Di Stefano, Giorgio De Conti, Raffaella Motta, Valeria Pergola

**Affiliations:** 1 Cardiology Unit, Cardio-thoraco-vascular and Public Health Department, Padova University Hospital, 35121 Padova, Italy; gabriele.cordoni@studenti.unipd.it (G.C.); filippo.amato@studenti.unipd.it (F.A.); eleonora.lassandro@studenti.unipd.it (E.L.); donatella.tansella@studenti.unipd.it (D.T.); diana.dipaolantonio@studenti.unipd.it (D.D.P.); mariamaddalena.baroni@studenti.unipd.it (M.M.B.); antonio.distefano.1@studenti.unipd.it (A.D.S.); valeria.pergola@unipd.it (V.P.); 2 Cardio-thoraco-vascular and Public Health Department, Padova University Hospital, 35121 Padova, Italy; morena.deamicis@studenti.unipd.it (M.D.A.); raffaella.motta@unipd.it (R.M.); 3 Emergency Institute for Cardiovascular Diseases and Transplantation of Targu Mures, 540136 Targu Mures, Romania; dan.alexandru03@yahoo.com; 4 Radiology Unit, Azienda Ospedale-Università Padova, 35121 Padova, Italy; simone.corradin@aopd.veneto.it (S.C.); stefano.dapozzo@aopd.veneto.it (S.D.P.); giorgio.deconti@aopd.veneto.it (G.D.C.)

**Keywords:** coronary artery calcium, perivascular fat attenuation index, coronary artery disease, cardiac computed tomography angiography, primary prevention

## Abstract

Coronary artery disease (CAD) is the leading global cause of mortality, accounting for approximately 30% of all deaths. It is primarily characterized by the accumulation of atherosclerotic plaques within the coronary arteries, leading to reduced blood flow to the heart muscle. Early detection of atherosclerotic plaques is crucial to prevent major adverse cardiac events. Notably, recent studies have shown that 15% of myocardial infarctions occur in patients with non-obstructive CAD, underscoring the importance of comprehensive plaque assessment beyond merely identifying obstructive lesions. Cardiac Computed Tomography Angiography (CCTA) has emerged as a cost-effective and efficient technique for excluding obstructive CAD, particularly in patients with a low-to-intermediate clinical likelihood of the disease. Recent advancements in CCTA technology, such as improved resolution and reduced scan times, have mitigated many technical challenges, allowing for precise quantification and characterization of both calcified and non-calcified atherosclerotic plaques. This review focuses on two critical physiological aspects of atherosclerotic plaques: the burden of calcifications, assessed via the coronary artery calcium score (CACs), and perivascular fat attenuation index (pFAI), an emerging marker of vascular inflammation. The CACs, obtained through non-contrast CT scans, quantifies calcified plaque burden and is widely used to stratify cardiovascular risk, particularly in asymptomatic patients. Despite its prognostic value, the CACs does not provide information on non-calcified plaques or inflammatory status. In contrast, the pFAI, derived from CCTA, serves as an indirect marker of coronary inflammation and has shown potential in predicting adverse cardiac events. Combining both CACs and pFAI assessment could offer a comprehensive risk stratification approach, integrating the established calcification burden with novel inflammatory markers to enhance CAD prevention and management strategies.

## 1. Introduction

Coronary artery disease (CAD) is the leading global cause of mortality, determining around 30% of total deaths [1]. CAD is characterized by the accumulation of atherosclerotic plaques in the coronary arteries, leading to slow narrowing or fast vessel obstruction and reduced blood flow to the heart muscle. Atherosclerotic plaque formation inside the arteries is the result of a complex interaction between lipoproteins, the endothelium, and inflammatory cells [2].

Since Russell Ross’s pioneering work, vascular inflammation has been recognized as a causal factor in the pathogenesis of all stages of atherosclerosis and plaque rupture [3]. Ross’s canonical response-to-injury hypothesis suggested that endothelial dysfunction—induced by factors such as genetics, vascular injury, elevated low-density lipoprotein cholesterol levels, free radicals from cigarette smoking, and hypertension—initiates atherosclerosis through collagen exposure and subsequent platelet adhesion, aggregation, and degranulation. Inflammation plays a critical role not only in the progression of atherosclerotic disease but also as a key driver of vulnerable plaque rupture, a primary pathogenic mechanism of myocardial infarction (MI).

Recent studies have shown that 15% of MIs occur in patients with non-obstructive CAD, highlighting the importance of comprehensive plaque assessment beyond detecting obstructive lesions [4]. Moreover, the International Study of Comparative Health Effectiveness With Medical and Invasive Approaches (ISCHEMIA) trial demonstrated that the traditional stenosis-based approach fails to identify at-risk patients and does not improve clinical outcomes beyond symptom relief in patients with stable CAD [5]. Therefore, identifying imaging markers specific to vascular inflammation is essential for the early detection of coronary inflammation before plaque formation becomes detectable.

Cardiac Computed Tomography Angiography (CCTA) has emerged as a cost-effective and efficient technique for excluding obstructive CAD. According to the last European Society of Cardiology Guidelines, CCTA is recommended to rule out CAD in patients with low-to-intermediate clinical likelihood, given its high negative predictive value [6]. This method is particularly useful for excluding significant atherosclerosis in patients with a low cardiovascular risk profile and typical or atypical symptoms, especially when obstructive CAD cannot be excluded by clinical assessment alone. CCTA has recently shown to be useful both in diagnosing the extent of CAD as well as excluding other causes of acute coronary syndrome [7,8,9].

Recent technological advancements have addressed technical challenges, such as the reduced temporal resolution of 64-section computed tomography (CT) detectors, with dual-tube systems or whole-heart coverage detectors, and beam artifacts for calcifications using spectral CT techniques [9]. Consequently, limitations of performing CCTA in patients with extensive coronary calcification, irregular heart rates, and significant obesity have been overcome [10].

Dedicated semi-automated and automated tools now enable precise quantification and characterization of various types of atherosclerotic plaques, including both calcified and non-calcified components [11,12]. CCTA can assess specific plaque features, such as low attenuation plaque, spotty calcification, positive remodeling, and the “napkin ring sign”, identifying the high-risk plaque phenotype that predicts major adverse cardiovascular events (MACEs) and plaque rupture. Conversely, calcification indicates stability and low inflammation.

In this review, we focus on two pivotal physiological aspects of atherosclerotic plaque: the burden of calcifications, assessed via the coronary artery calcium score (CACs), and pericoronary adipose tissue (PCAT) inflammation, measured through the perivascular fat attenuation index (pFAI). Both the CACs and pFAI, obtained from CCTA acquisitions using post-processing software, represent radiological expressions of complex pathophysiological processes. They provide non-invasive methods for studying these processes, representing two sides of the same coin. This review emphasizes the importance of integrating these radiological markers to enhance the assessment and management of CAD while providing an overview of the key studies available in the literature.

## 2. Coronary Calcium Score

### 2.1. Definition

The CACs measures the amount of calcium in the walls of the coronary arteries using non-enhanced CT scans. The presence of calcium in the coronary arteries is a specific marker of subclinical atherosclerosis. In particular, the CACs provides an accurate measurement of the coronary calcific plaque burden, as confirmed by previous histopathological studies [13].

### 2.2. Measurement

The CACs is obtained through a non-contrast CT scan of the heart. Data are acquired using prospective ECG triggering in late diastole (70–80% R-R interval) and a 120 kV tube voltage. The CACs is typically computed using the Agatston method, which considers plaque density scores. The CACs is calculated by multiplying the calcified plaque area by the density score. The calcified plaque area is identified by the reader, and the density of each region of interest is determined by selecting the voxel with the highest density, and thus, the highest X-ray attenuation. Only lesions with an area of at least 1 mm^2^ are counted, eliminating single pixels with attenuation >130 Hounsfield Unit (HU), which are likely artifacts. Lesion areas are automatically measured in square millimeters, and the maximum HU of each region of interest is recorded. An arbitrary weighting factor is applied in the calculation based on the maximal HU [14].

**Figure 1:**
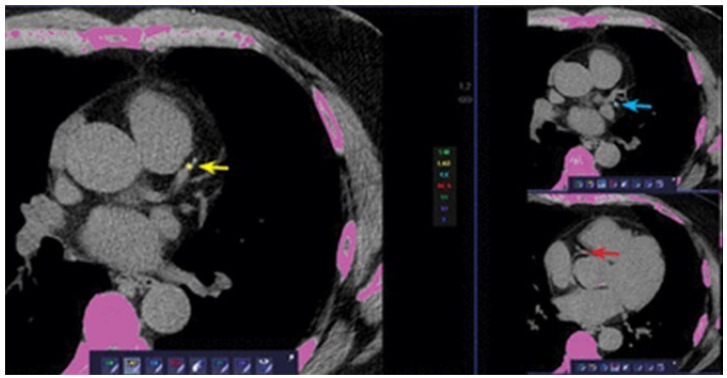
CAC score assessment. The provided image illustrates a calcium score evaluation of the coronary arteries. The yellow arrow highlights the calcium score measurement on the left anterior descending coronary artery. The red arrow indicates the calcium score measurement on the right coronary artery. Lastly, the blue arrow points to the calcium score measurement on the circumflex coronary artery.

The total result (total Agatston score, or more commonly, the CACs) is obtained by summing the scores for all the lesions in all coronary arteries (Figure 1). The CACs can be reported either as an absolute value or as a percentile in comparison to age-, sex-, and ethnicity-matched individuals [15].

The CACs risk categories provide an estimation of the probability of severe CAD, as summarised in Table 1.

### 2.3. Prognostic Value

Several prospective large-scale trials have addressed the prognostic value of CACs with data derived from population and clinical cohorts [Multi-Ethnic Study of Atherosclerosis (MESA) [16]; Framingham Heart Study [17]; the Dallas Heart Study [18]; BioImage [19] and the Heinz Nixdorf Recall study [20]]. These studies have established the CACs as a valuable tool in stratifying cardiovascular risk, especially in asymptomatic patients (Table 2). In primary prevention, the CACs, combined with clinical evaluation, is widely used to further stratify cardiovascular risk in asymptomatic individuals. This is particularly important for intermediate-risk individuals where preventive cardiovascular treatment decisions are often uncertain [21]. The CACs can reclassify cardiovascular disease (CVD) risk both upwards and downwards in addition to conventional risk factors, and it is especially useful when calculated risk is near decision thresholds. A higher CACs indicates a greater burden of calcified plaque, which is associated with an increased risk of MI and death [21].

The Expert Consensus Statement from the Society of Cardiovascular Computed Tomography highlights the role of the CACs in defining therapeutic strategies for asymptomatic patients. For those with a low 10-year arteriosclerotic cardiovascular disease (ASCVD) risk (<10%), a low CACs can reassure clinicians of a low cardiovascular event risk, whereas a higher CACs may prompt a reassessment of the current therapy. In patients with an intermediate 10-year ASCVD risk (10–20%), the CACs is crucial for determining clinical risk. Very low CAC values can downgrade risk, while high CAC values can upgrade it. For patients with high ASCVD risk, the CACs is not indicated, as these patients are already considered high risk by definition [22].

Discrepancies between the presence of CAC and traditional risk factors may influence the decision to start preventive drug treatment. The CAC score can reclassify up to 50% of patients with an intermediate Framingham score to either low or high risk, better-identifying patients appropriate for statins [23].

The Risk Or Benefit IN Screening for CArdiovascular disease (ROBINSCA) trial [24], a population-based randomized controlled screening trial, compared the Dutch Systematic Coronary Risk Evaluation (SCORE) risk model, which predicts a 10-year risk for developing fatal and non-fatal CVD, with the CACs as different potential screening modalities. The trial found that CACs identified more low-risk individuals compared to the SCORE model and classified significantly fewer individuals at intermediate and high risk. This suggests that CACs can reduce preventive overtreatment and associated stress from receiving an unfavorable test result.

Additionally, the CorCal study (Effectiveness of a Proactive Cardiovascular Primary Prevention Strategy, With or Without the Use of Coronary Calcium Screening, in Preventing Future Major Adverse Cardiac Events) [25] compared two proactive ASCVD primary prevention strategies: CAC-guided (investigational) versus pooled cohort equation-guided (standard) statin therapy. The study confirmed that the CAC-guided strategy reclassified statin treatment recommendations for a substantial percentage of patients, with more individuals being reclassified to no statin therapy, thereby reducing overtreatment as demonstrated in the ROBINSCA trial.

**Table 2:** Summary of several major large-scale prospective trials, all of which have investigated the prognostic value of the CACs in cardiovascular risk assessment. A literature review of main studies exploring the role of CAC score in assessing cardiovascular risk and coronary heart disease. CVD: cardiovascular disease; CAC: coronary artery calcium; CAD: coronary arteries disease; CV: cardiovascular; FU: follow-up.

Study/Work	Year	Country	*n*. Patient	Age	FU	Main Findings	Journal
South Bay Heart Watch [26]	2004	USA	1312	65.7	8.5 years	CAC score significantly modifies risk prediction in all patients with moderate CV risk	*Journal of American Medical Association*
Coronary Artery Calcium Score and Coronary Heart Disease Events in a Large Cohort of Asymptomatic Men and Women [27]	2005	Dallas	16,097	53.8 years	3.5 years	CAC score predicts subclinical atherosclerosis and CAD	*American Journal of Epidemiology*
PACC Project [28]	2005	Washington	1983	42.8	3 years	CAC score to better stratify patients at moderate CV risk	*Journal of the American College of Cardiology*
MESA sub-analysis [16]	2008	USA	6722	45–84 years	3.8 years	CAC score in multi-ethnic populations predicts CAD over standard coronary risk factors	*New England Journal of Medicine*
All-cause mortality by age and gender based on coronary artery calcium scores [29]	2016	USA	13,092	58 ± 11 years	15 years	CAC score in patients >75 years has a low predictive power of mortality risk for higher rate of non-cardiac death. CAC = 0, regardless of risk factors, correlates with very low long-term mortality	*European Heart Journal—Cardiovascular Imaging*
Incidental coronary artery calcification on non-gated CT thorax correlates with risk of cardiovascular events and death [30]	2022	England	717	40–70 years	41.6 months	Incidental CAC correlates short-term CVD and deaths	*European Radiology*
Heinz Nixdorf Recall [31]	2023	Essen, Germany	4154		20 years	CAC score stratifies in 20 years FU better than assessing CV risk factor, especially in patients initially at intermediate risk	*Deutsches Ärzteblatt International*

### 2.4. Clinical Guidelines

In clinical practice, the CACs is particularly valuable for asymptomatic individuals, helping to guide preventive strategies and treatments based on the level of patient risk. For asymptomatic patients with an intermediate risk of cardiovascular events, American guidelines suggest adjusting lipid-lowering therapy based on the CACs, if available [32,33]. Specifically, if the CACs is 0, it is possible to discontinue or not initiate lipid-lowering therapy, with re-evaluation of the patient in 5–10 years or if new cardiovascular risk factors emerge, unless other risk factors, such as family history of CVD, smoking, or diabetes, are present. For patients aged 55 years or older with a CACs between 1 and 99, initiating lipid-lowering therapy should be considered. If the CACs exceeds 100, lipid-lowering therapy may be indicated regardless of age [34].

A recent review [34] focused on the recommendations for the use of the CACs according to international worldwide guidelines. Several common points emerged where different guidelines agree, such as the recommendation to assess the CACs in asymptomatic patients over 40 years old who are at intermediate cardiovascular risk.

Regarding lipid-lowering therapy, it is acknowledged that a CACs of 0 is sufficient to downgrade the patient’s risk and potentially discontinue lipid-lowering therapy. Conversely, for CACs greater than 100, there is a consensus on the indication to initiate or consider statin therapy based on the patient’s risk profile.

When considering aspirin therapy guided by the CACs, international guidelines diverge due to the concern of bleeding risk, which limits the indication to single antiplatelet therapy. Utilizing meta-analysis data on aspirin’s impact on CVD relative risk reduction and bleeding risk, Cainzos-Achirica et al. [35], Miedema et al. [36], and Greenland et al. [37] each conclude that the CACs can identify sub-cohorts of individuals who may benefit from aspirin therapy. For subgroups with CACs greater than 100, especially those with a CACs over 400, aspirin provides a net benefit regardless of other risk factors. However, for individuals with a CACs of 0, the bleeding risk outweighs the potential benefits of aspirin. Table 3 summarizes the role of the CACs in clinical practice, according to the main international guidelines.

### 2.5. Limitations

The use of the CACs has several notable limitations. One primary limitation is its inability to detect non-calcified plaques, which are significant predictors of coronary events. While the CACs effectively marks anatomical disease and strongly correlates with incident vascular events, it fails to identify lipid-laden, rupture-prone “soft plaques”, which are regarded as the primary precursors of CAD events [38].

Furthermore, 1% to 2% of patients, particularly younger individuals (women < 55 years and men < 45 years) with a zero CACs may still have obstructive CAD due to the presence of non-calcified plaques. This underscores the CACs’s limitation as an imperfect marker of CAD risk, as it can miss non-calcified plaques [39].

A previous study by our group [40] further highlighted these limitations. We found that while the CACs was positively related to in-hospital mortality in COVID-19 patients, it did not completely identify all individuals at risk for adverse events. This suggests that other factors, such as the presence of soft, unstable plaques, play a significant role in adverse outcomes.

## 3. Perivascular Fat Attenuation Index

### 3.1. Definition

CCTA provides a non-invasive method for directly imaging vascular inflammation, which enhances its potential for widespread clinical use. Its current inclusion in clinical guidelines for investigating chest pain worldwide underscores its established utility. Traditionally, coronary plaque risk is stratified based on plaque stability to assess the likelihood of cardiac events. A new CCTA-derived imaging biomarker, the pFAI, has shown promise in this area. The pFAI can detect phenotypic changes in PCAT and serve as an indicator of vascular inflammation by identifying gradients in PCAT attenuation. This development offers a newer understanding of vascular health and could improve risk stratification in clinical practice [41].

### 3.2. Measurement

PCAT serves as a sensitive indicator of inflammatory signals from the vascular wall, transforming adipocytes into active biosynthetic cells through the activation of peroxisome proliferator-activated receptor gamma (PPAR-γ) and reducing their lipid content [42]. These transformed adipocytes secrete antioxidant adipokines like adiponectin to protect the vascular wall from oxidative damage and inhibit the differentiation of pre-adipocytes while promoting perivascular lipolysis, creating a gradient in adipocyte size [42].

This gradient in lipid/water ratio around the inflamed artery can be visualized and quantified from routine CCTA as a gradient in CT signal attenuation in the peri-vascular space [43]. The pFAI leverages this principle, detecting inflammation-induced changes in adipocyte size associated with a shift in CT attenuation toward a less negative HU range (closer to −30 HU). Therefore, in inflamed coronary arteries, the pFAI shifts from more negative values (around −190 HU) to less negative values (closer to −30 HU). Higher pFAI values are indicative of greater inflammatory burden [43].

**Figure 2:**
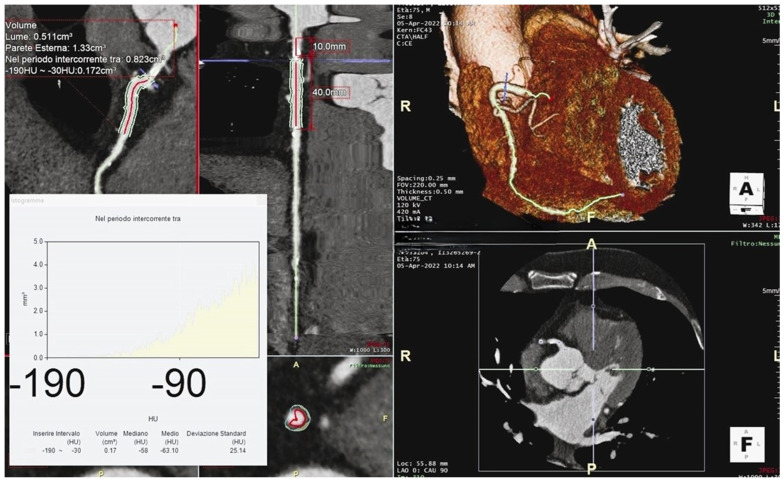
The image depicts the evaluation of the pFAI around the proximal segment of the right coronary artery (RCA). The pFAI measurement was taken over a 40 mm segment (10–50 mm from the RCA origin) at a radial distance equal to the artery’s diameter, with the most proximal 10 mm segment excluded to avoid the effects of the aortic wall. The highlighted median value indicates an inflamed coronary artery.

The pFAI is defined as the weighted mean attenuation of all adipose tissue-containing voxels (−190 to −30 HU) within a radial distance from the outer vessel wall equal to the diameter of the relevant vessel. To avoid the effects of the aortic wall, the most proximal 10 mm segment of vessel is excluded. Initially, the pFAI was measured around the proximal segment of the right coronary artery (RCA) over a 40 mm segment (10–50 mm from the RCA origin) at a radial distance equal to the artery’s diameter (Figure 2). Further validation studies have led to the development of algorithms to calculate the pFAI around the proximal segments of the left circumflex artery (LCA) and the left anterior descending artery (LAD) [44,45].

### 3.3. Prognostic Value

PCAT attenuation serves as an indirect marker of coronary inflammation, offering valuable insights into cardiovascular risk stratification. Studies by Lin et al. and Goeller et al. have demonstrated that increased pFAI values correlate with different stages of CAD and higher PCAT attenuation around culprit lesions in acute coronary syndrome, underscoring its potential to track local inflammatory changes [46,47]. This indicates that pFAI could reflect both vascular inflammation and luminal stenosis, suggesting a nuanced role in coronary event prediction.

Moreover, the CRISP-CT study highlighted that elevated pFAI values around the proximal RCA and LAD are strong predictors of all-cause and cardiac mortality, showing its prognostic utility in these coronary segments [41]. While the predictive power of the pFAI varies across different coronary segments, its ability to forecast adverse cardiac events in key arteries is significant.

The development of advanced software like CaRi-HeartVR, which combines clinical risk factors, coronary plaques, and inflammation data, represents a significant leap forward in personalized cardiac risk estimation, promising improved risk stratification and treatment guidance compared to traditional methods [45]. A previous study highlighted that a higher pFAI (≥70.1 HU) was significantly associated with increased risks of MACE, in patients with non-severe coronary plaques. Patients with an elevated pFAI had greater overall mortality and required more interventional procedures, independent of other risk factors. This underscores the importance of the pFAI as a valuable CCTA-derived marker for identifying higher-risk patients who might benefit from more aggressive therapeutic management and closer follow-up, despite having non-severe, non-calcified plaques [11].

In the context of MI with non-obstructive coronary arteries, our group reported that RCA pFAI values in patients with clinically suspected myocarditis were lower than those typically found in obstructive atherosclerosis [48]. This distinction likely reflects different underlying mechanisms of coronary inflammation in myocarditis versus CAD. The study concluded that in clinically suspected myocarditis with infarct-like presentation, higher pFAI values correlated with higher biventricular end-systolic volume and lower right ventricular ejection fraction, suggesting a role for the pFAI in predicting non-atherosclerotic coronary inflammation, such as infective or immune-mediated endothelialitis.

Lastly, the pFAI as a dynamic marker of PCAT phenotype changes has shown promise in tracking the effects of lipid-lowering treatments. Goeller et al. observed that changes in the pFAI correlated with changes in non-calcified plaque burden over a follow-up period, indicating that the pFAI might precede plaque formation [49]. Mátyás et al. investigated the long-term effects of high-dose lipid-lowering therapy over a 3-year period on the pFAI in patients with low to intermediate probability of CAD. The study observed significant morphological changes in coronary plaques, noting an increase in calcified components within mixed plaques and a reduction in non-calcified plaque volume. Notably, PCAT attenuation decreased after 1 year of therapy and remained low thereafter, indicating a sustained reduction in arterial inflammation across all coronary arteries [50]. However, the reliance on the pFAI as an endpoint in clinical trials necessitates further research to establish its role compared to circulating plasma biomarkers, which are not specific for vascular inflammation. Recently, a comprehensive model combining clinical risk factors with radiomic features derived from the PCAT surrounding major coronary arteries was developed to predict MACE within 3 years. This integrated model demonstrated superior predictive performance for MACE compared to models based solely on clinical factors or radiomic features. Additionally, the inclusion of PCAT data from all three major coronary arteries resulted in higher predictive accuracy than models focusing on a single coronary. Among these, the PCAT surrounding the RCA showed the best performance. These findings highlight the essential role of the pFAI in cardiovascular risk assessment and suggest that its diagnostic accuracy can be further enhanced by incorporating traditional clinical risk factors [51].

### 3.4. Limitations

The primary limitation of using the pFAI as a prognostic indicator is the lack of extensive validation in large, diverse populations. Moreover, the assessment of the pFAI necessitates advanced imaging techniques and specialized expertise, making it less accessible compared to simpler measures such as the CACs. The clinical interpretation of pFAI values is influenced by various technical factors, including tube voltage and contrast media, as well as the specific anatomical characteristics of the coronary artery segment being analyzed. Furthermore, biological factors such as patient demographics (age, gender, and obesity) also play a critical role in the interpretation of pFAI values [52].

## 4. Comparative Summary

**Figure 3:**
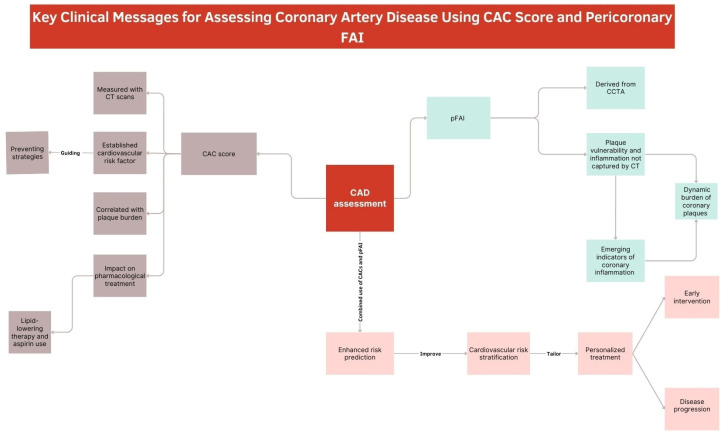
This graphic summarizes the roles of the pFAI and CAC score in assessing coronary artery disease risk. By integrating both parameters, cardiovascular risk stratification is enhanced, enabling more personalized patient therapy.

Over the last few decades, different imaging modalities have been introduced in the field of multi-modality imaging, aiming at better understanding the morpho-functional abnormalities occurring in cardiovascular diseases [53]. CACs and pFAI assessment can be considered as the radiological expression of two different physio-pathological moments (Figure 3).

On one hand, CACs reflect a non-reversible process of coronary calcification that typically does not regress and may increase despite appropriate medical treatments like statins. It is primarily useful for identifying the burden of calcified plaques, marking anatomical disease, and correlating strongly with incident vascular events. However, it is limited in secondary prevention due to its inability to reflect changes in non-calcified plaque burden.

On the other hand, the pFAI provides a measure of local coronary inflammation, which plays a critical role in both the formation of atherosclerotic plaques and their rupture, leading to acute coronary syndrome. It could be considered a modifiable risk factor. Non-invasive detection of residual inflammatory coronary risk via the pFAI can facilitate more timely preventive measures in primary care and the effect of these measures could be evaluated through repeat CT scans. The pFAI has been shown to correlate with CAD independently of CACs, age, gender, cardiovascular risk factors, and the atherosclerotic plaque burden [43].

In the context of obstructive CAD, an elevated CACs is typically associated with a high calcific plaque burden, suggesting a stable, chronic obstructive condition. However, obstructive non-calcified plaques are not detected by the CACs. Conversely, elevated pFAI values are more indicative of plaque vulnerability, regardless of the type of plaque (calcified, non-calcified, or mixed). This distinction is important because inflammation often precedes acute events involving especially non-obstructive plaques. According to Lin et al., patients with MI exhibited significantly higher PCAT attenuation (−82.3 ± 5.5 HU) compared to those with stable CAD (−90.6 ± 5.7 HU, *p* < 0.001) and healthy controls (−95.8 ± 6.2 HU, *p* < 0.001) [46].

### 4.1. Clinical Implications

In clinical practice, CACs is extensively utilized and have been integrated into global guidelines for cardiovascular risk assessment due to its ease of measurement, interpretability, and well-documented clinical significance. Its application is particularly pertinent in primary prevention, where it serves as a decisive tool in refining cardiovascular risk stratification, especially in borderline cases. By enabling the reclassification of patients’ risk—either downgrading or upgrading their risk category—the CACs facilitates the customization of preventive therapy tailored to the individual, irrespective of traditional risk factors. This underscores its pivotal role in guiding clinical decision-making beyond conventional risk assessments.

As an emerging biomarker, the pFAI provides additional insights into local coronary inflammation and may enhance risk prediction when used alongside traditional methods. As in the presence of other classical high-risk plaque features—such as low attenuation plaque, spotty calcification, positive remodeling, and the “napkin ring sign”—an elevated pFAI significantly increases the risk of MACE. This heightened risk necessitates a more aggressive preventive strategy, which may include intensified lipid-lowering therapy and the use of anti-inflammatory medications, both of which have demonstrated benefit in several studies [54,55]. However, its clinical utility is still being explored, and its assessment is more technically demanding so its use is not accepted worldwide.

**Figure 4:**
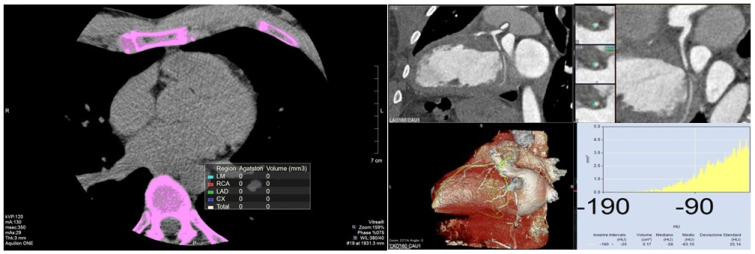
A 45-year-old male with a history of hypertension and hyperlipidemia presents for emergency evaluation. The patient is symptomatic for chest pain. A coronary computed tomography angiography was performed. The right image shows a coronary artery calcium score = 0 for all coronary arteries, indicating no detectable calcified plaque. In the left panel, the Multiplanar Reformation and Volume Rendering show a clear soft plaque on left circumflex artery. The elevated perivascular fat attenuation index indicates a vulnerable plaque. These findings suggest the absence of calcified plaque but the presence of a soft, potentially vulnerable plaque that may predispose the patient to future cardiovascular events.

Our review stands out by providing a comprehensive, literature analysis of both established and emerging markers of atherosclerotic plaque burden and inflammation. Specifically, by integrating the CACs and pFAI, old and novel biomarkers, this integrative approach not only bridges the gap between traditional calcification metrics and novel inflammatory indicators but also enhances the potential for early detection and personalized treatment strategies (Figure 4). By combining these assessments, our review aims to advance the field by providing a more nuanced understanding of cardiovascular risk, ultimately contributing to improved prevention and management of CAD.

### 4.2. Limitation

The absence of information about non-calcified plaques or inflammatory status is the main CACs limitation, potentially missing significant predictors of coronary events. Although the pFAI could overcome these limits, requires advanced imaging techniques and specialized expertise, as it is influenced by a range of technical factors, anatomical characteristics, and biological ones. Table 4 summarizes the pros and cons of CACs and pFAI analysis.

To overcome these limits, future studies should include larger and more diverse populations to reduce potential biases and enhance the generalizability of the findings. Conducting longitudinal studies could provide more insight into the progression and impact of CAC and pFAI over time and incorporating advanced imaging methods could help in obtaining more precise measurements and reduce potential errors related to current techniques. Lastly, investigating the effects of specific interventions on CAC and pFAI could provide practical insights into their management and treatment.

## 5. Conclusions

Incorporating both the CACs and pFAI into clinical practice could revolutionize cardiovascular risk assessment. The CACs, with its established measure of calcification burden, and the pFAI, with its insights into coronary artery inflammation, together provide a more precise risk stratification, enabling more personalized and effective preventive strategies. Ultimately, the integration of these metrics can significantly improve patient outcomes by identifying high-risk individuals more accurately and tailoring interventions accordingly.

## Figures and Tables

**Table 1:** CAC score categories and their associated risk levels.

CAC Score	Risk Category	Probability of Severe CAD
0	Very low risk	<5%
1–99	Mildly increased	5–10%
100–299	Moderately increased	10–20%
300–1000	Moderate to severely increased	20–40%
>1000	Severely increased	>40%

**Table 3:** Summarizes the clinical aspects related to the prognostic value of the CACs. CACs: coronary artery calcium score; CVD: cardiovascular disease; ASCVD: arteriosclerotic cardiovascular disease.

Role	Details
**Risk Stratification**	Used in primary prevention, especially in asymptomatic individuals Crucial for intermediate-risk individuals to guide treatment decisions Reclassifies CVD risk upwards or downwards in addition to conventional risk factors
**Impact on Therapy**	Low CACs in low ASCVD-risk individuals can reassure clinicians High CACs may prompt a therapy reassessment Not indicated for patients already high ASCVD risk by definition
**Guidelines for Lipid-Lowering Therapy**	CACs = 0: possible discontinuation or avoidance of lipid-lowering therapy, unless other risk factors are present CACs = 1–99: consider initiating lipid-lowering therapy, particularly in patients aged 55+ CACs > 100: likely indication for therapy
**Guidelines for Aspirin Therapy**	CACs > 100: aspirin may provide a net benefit, especially with scores > 400 CACs = 0: bleeding risk outweighs benefits, suggesting aspirin may not be indicated

**Table 4:** Pros and cons of coronary artery calcium score and pFAI. CACs: coronary artery calcium score, CAD: coronary artery disease.

Feature	CAC Score	pFAI
**Pros**	Widely used and accepted in clinical practice	Provides insights into local coronary inflammation
	Simple to measure and interpret	Can detect residual inflammatory coronary risk
	Strong correlation with incident vascular events	Correlates with CAD independently of CACs, age, gender, risk factors
**Cons**	Does not detect non-calcified plaques	Requires advanced imaging techniques and specialized expertise
	Limited in secondary prevention	Influenced by technical, anatomical, and biological factors
		Clinical utility still being explored

## Data Availability

No new data were created or analyzed in this study. Data sharing is not applicable to this article.

## Abbreviations

ASCVD: arteriosclerotic cardiovascular disease; CACs: coronary artery calcium score; CAD: coronary artery disease; CCTA: cardiac computed tomography angiography; CVD: cardiovascular disease; CT: computed tomography; HU: Hounsfield unit; LAD: left anterior descending; LCA: left circumflex artery; MACE: major adverse cardiac events; MESA: Multi-Ethnic Study of Atherosclerosis; MI: myocardial infarction; PCAT: peri-coronary adipose tissue; p-FAI: perivascular fat attenuation index; RCA: right coronary artery; ROBINSCA: Risk or Benefit IN Screening for Cardiovascular disease; SCORE: Systematic Coronary Risk Evaluation.
